# The Regulation of Muscle Structure and Metabolism by Mio/dChREBP in *Drosophila*

**DOI:** 10.1371/journal.pone.0136504

**Published:** 2015-08-25

**Authors:** Grzegorz L. Polak, Anthony Pasqualino, James E. B. Docherty, Stephen J. Beck, Justin R. DiAngelo

**Affiliations:** 1 Department of Biology, Hofstra University, Hempstead, NY, 11549, United States of America; 2 Department of Biology, Nassau Community College, Garden City, NY, 11530, United States of America; 3 Division of Science, Penn State Berks, Reading, PA, 19610, United States of America; University of Valencia, SPAIN

## Abstract

All cells require energy to perform their specialized functions. Muscle is particularly sensitive to the availability of nutrients due to the high-energy requirement for muscle contraction. Therefore the ability of muscle cells to obtain, store and utilize energy is essential for the function of these cells. Mio, the *Drosophila* homolog of carbohydrate response element binding protein (ChREBP), has recently been identified as a nutrient responsive transcription factor important for triglyceride storage in the fly fat body. However, the function of *Mio* in muscle is unknown. In this study, we characterized the role of Mio in controlling muscle function and metabolism. Decreasing *Mio* levels using RNAi specifically in muscle results in increased thorax glycogen storage. Adult Mio-RNAi flies also have a flight defect due to altered myofibril shape and size in the indirect flight muscles as shown by electron microscopy. Myofibril size is also decreased in flies just before emerging from their pupal cases, suggesting a role for Mio in myofibril development. Together, these data indicate a novel role for Mio in controlling muscle structure and metabolism and may provide a molecular link between nutrient availability and muscle function.

## Introduction

Skeletal muscle comprises over one-third of the body mass of a healthy individual and is responsible for 20 to 30 percent of the body’s overall basal metabolic rate [1]. Alterations of skeletal muscle structure or its metabolism can lead to a number of diseases. Muscular dystrophies have been shown to result from mutations in genes coding for muscle structural proteins, the most common being dystrophin. Dystrophin functions to anchor sarcolemmal proteins to the cytoskeleton and loss of this protein from the sarcolemmal membrane results in necrosis of the muscle fibers [2, 3]. Muscle weakness has also been observed in cancer [4] and metabolic diseases such as diabetes [5], presenting a link between metabolism and muscle function. Therefore, expanding our knowledge of the mechanisms controlling the development and function of muscle will be important to further our understanding of the pathogenesis of these diseases.

Mammalian skeletal muscle relies heavily on oxidative phosphorylation for energy and utilizes glucose as the primary energy source [6]. In order to obtain glucose for energy, insulin acts on mammalian skeletal muscle leading to an increase in glucose uptake and oxidation [7]. In addition to acting as metabolic substrates, glucose and its metabolites also act as signaling molecules that control cell physiology [8]. One molecule that responds to changes in glucose concentration in the mammalian liver is carbohydrate response element binding protein (ChREBP). ChREBP, together with its binding partner Mlx, controls glucose-induced gene expression by binding to promoters of target genes, many of which are important for glucose utilization and triglyceride storage [9]. ChREBP is expressed in a number of tissues, with high levels found in mammalian liver, adipose tissue, skeletal muscle and intestine [10]. ChREBP also regulates the expression of important glycolytic enzymes such as pyruvate kinase (Pyk) and genes that encode enzymes important for fatty acid synthesis such as fatty acid synthase (FAS) and acetyl-CoA carboxylase (ACC) [10, 11]. Another transcription factor that regulates gene expression in response to glucose is MondoA. MondoA is expressed in many tissues but is highly enriched in skeletal muscle [12]. Like ChREBP, MondoA also binds to Mlx forming a heterodimer to control gene expression [13]. However, unlike ChREBP, the MondoA-Mlx complex associates with the outer mitochondrial membrane and shuttles between the mitochondria and the nucleus where it activates the transcription of genes encoding glycolytic enzymes such as 6-phosphofructo-2-kinase, fructose- 2,6-bisphosphatase and hexokinase II [9, 11, 14].

While the role of ChREBP has been well characterized in liver and in pancreatic β-cells, its function in other tissues is not well understood. ChREBP has been shown to regulate glucose-induced expression of glycolytic enzymes in cultured myotubes [15]; however, the *in vivo* function of ChREBP in muscle remains unknown. To better understand the function of ChREBP in muscle, we took advantage of the model organism *Drosophila melanogaster*. *Drosophila* contains a single ChREBP/MondoA homolog called Mio [16, 17]. Mio/dChREBP acts in the *Drosophila* fat body to regulate sugar-induced gene expression as well as triglyceride storage [18–20], showing high conservation of ChREBP function between flies and mammals.

*Drosophila* is an ideal system to study muscle function as flies have muscle groups with similar morphology and physiology to mammalian skeletal muscle. One example of these muscles is those necessary for commencing and sustaining flight—the direct and indirect flight muscles (DFM and IFM, respectively). Defects in the ultrastructure of DFM or IFM cause flightless or impaired flight phenotypes that do not lead to lethality of the animal. Consequently, a number of genes important for muscle structure, assembly and maintenance, such as myosin heavy chain (Mhc), Actin88F (Act88F), flightin and kettin, which are similar to the mammalian elastin and titin, respectively, have been identified using the *Drosophila* system [21–27]. Like mammalian skeletal muscle, *Drosophila* IFM is also highly metabolic requiring large amounts of energy for flight. *Drosophila* IFM and DFM use oxidative phosphorylation for energy similar to mammalian skeletal muscle. Consistent with the importance of glycolysis for energy production, some glycolytic enzymes co-localize on the Z-line of the sarcomere suggesting that they play an important role in energy delivery associated with insect flight [28, 29]. While many genes important for *Drosophila* muscle structure and development have been identified, the full complement of genes that regulate muscle function and metabolism are still unknown.

In this study, we characterized Mio function in *Drosophila* muscle. Decreasing Mio specifically in muscle using RNAi leads to increased glycogen storage. We also show that loss of Mio results in irregular myofibril assembly and decreased myofibril area decreasing flight ability. Together, these studies identify a novel role for Mio in regulating muscle structure and function and provide further support for the use of *Drosophila* as a model system for understanding the tissue-specific control of physiology and metabolism.

## Materials and Methods

### Fly Stocks

The UAS-GFP (#1522) and Mef2-Gal4 (#27390) lines were obtained from the Bloomington Stock Center. UAS-Mio-IR (#52606) was obtained from Vienna *Drosophila* RNAi center and activates RNA interference through the use of inverted repeats, while UAS-Mio^dsRNA^ triggers RNAi through the production of double stranded RNAs as described previously [20]. All flies were grown in 25°C, on a standard cornmeal-sugar-yeast medium.

### Glycogen and triglyceride quantification

Thoraxes from three or four 5–7 day old female or male flies, respectively, were homogenized in lysis buffer (140mM NaCl, 50mM Tris-HCl, pH 7.4, 0.1% Triton-X, and 1X protease inhibitor cocktail (Roche Diagnostics)) as described previously [30]. Glucose Oxidase reagent (Pointe Scientific) was used to measure free glucose concentrations from homogenized thoraxes as well as total glucose concentration from homogenized thoraxes treated with 8 mg/ml amyloglucosidase (Sigma) in 0.2M sodium citrate buffer, pH 5.0 for 2 hours at 37°C. To determine the final glycogen concentration, free glucose concentration was subtracted from the total glucose levels measured after amyloglucosidase treatment. Triglyceride and protein concentrations were measured using Stanbio Liquicolor (Fisher Scientific) and BCA protein Assay (ThermoScientific) kits, respectively according to manufacturers protocol. Glycogen and triglyceride data were normalized to protein levels by dividing the glycogen or triglyceride concentration by total protein concentration. The total protein concentrations were similar across all genotypes (data not shown).

### Flight Test

Flight tests were performed using a 1000 mL graduated cylinder coated with mineral oil adapted from [24]. Flight scores were assigned as previously described [24] with slight modifications. Flies were knocked into the top of the graduated cylinder through a funnel and given a score ranging from 0 (no flight ability) to 4 (full flight ability) depending on how high up the cylinder they were when they flew horizontally into the oil covered glass.

### Electron Microscopy

For Scanning and Transmission Electron Microscopy (SEM or TEM, respectively), we used females for all experiments due to their larger size (and therefore more flight muscle tissue per animal) and ease of dissection. Pharate adults or adult flies 6 days after eclosion were collected and placed directly into 3% glutaraldehyde in 0.1 M sodium-cacodylate or sodium phosphate buffer, pH 7.2 at 4°C followed by careful removal of the head and abdomen. Thoraxes remained in the fixative for 60 minutes followed by a wash in 0.1M sodium phosphate or sodium-cacodylate buffer, pH 7.2 at 4°C. Samples were post-fixed in 1% OsO_4_ in 0.1 M sodium phosphate or sodium-cacodylate buffer, pH 7.2 at 4°C for an additional 60 minutes and rinsed in 0.1 M phosphate or sodium-cacodylate pH 7.2 at 4°C. For TEM, samples were dehydrated in ethanol (70%, 95%, 100% x 2) followed by transition to propylene oxide. Samples were then embedded in an Epon 812/Araldite 6005 mixture in BEEM capsules (Electron Microscopy Sciences) or placed in flat molds which were sectioned either horizontally of transversely. The ultra-thin 70 nm transverse and horizontal sections were cut using a diamond or glass knife on a Sorvall MT-2B ultramicrotome and post stained with uranyl acetate followed by lead citrate [31]. Sections were visualized using an HS-8–2 TEM (Hitachi, Japan) at 50 kV and an FEI Quanta 250 scanning electron microscope (FEI, Czech Republic) at 30 kV.

For SEM, after fixation as described above, samples were dehydrated in ethanol (30%, 50%, 70%, 95%, 100%) followed by cryofracture in liquid nitrogen. Samples were critical point dried using a Samidri-795 dryer (Tousimis) and gold-coated using an EMS-550 sputter coater to 10nm coat thickness (Electron Microscopy Sciences). Samples were visualized using an FEI Quanta 250 scanning electron microscope (FEI, Czech Republic) at 30 kV.

### Myofibril area quantification

Using ImageJ software (http://rsbweb.nih.gov/ij), adult myofibril area was measured from SEM images from 3–5 individual flies per genotype, while myofibrils from pharate adults were measured from TEM images taken from 3–4 individual animals per genotype. At least three micrographs of cross sections were taken from each animal. 10–15 myofibrils were measured per micrograph to give a total of 60–120 myofibrils quantified, depending on the genotype. Average myofibril areas were calculated from all of the myofibrils quantitated from each genotype and analyzed as described below in the *Statistics* section.

### RNA isolation and quantitative PCR

RNA was isolated from thoraxes of 5–7 day old flies (10 females and 5 males per sample). Thoraxes were homogenized in Ribozol (Amresco) and extracted with chloroform. An equal amount of isopropanol was added and samples were incubated at 4°C for 10 min followed by centrifugation at 12,000 rpm for 15 min at 4°C. Pellets were washed twice with 70% EtOH and then resuspended in water. Genomic DNA was removed using the Turbo DNA-free kit (Applied Biosystems) according to manufacturers instructions. 0.25–1 μg of total RNA was reverse transcribed using the RETROscript kit (Ambion) according to manufacturers protocol using random decamers. Quantitative PCR was performed using Power SYBR Green MasterMix (Applied Biosystems) in a StepOnePlus thermocycler (Applied Biosystems). The expression of each experimental gene was normalized to the ribosomal protein gene, rp49. Primer sequences for qPCR are shown in Table 1.

**Table 1:** 

Gene Name	Forward (5’ to 3’)	Reverse (5’ to 3’)
Mio	AGCGAGACGAGCTAAACAATTC	GTGTAAGAGGCAAGCAAAGGTT
rp49	GACGCTTCAAGGGACAGTATCTG	AAACGCGGTTCTGCATGAG
Act88F	TGATGCGGGTGCATTAGTTA	ATGGGGTACTTCAGCGTCAG
MHC	CCGTGCGTAACGATAACTCC	ATGTGGTAGGAACGCTCCAG
Mlc-2	CAACACCACCAACCAGATCA	GTTCTTGCCGTGACAGTGAA

#### Statistics

All data was tested for normality in IBM SPSS 20.0 software using Shapiro-Wilk (n<50), and Kolmogrov-Smirnov (n>50) tests. The majority of the data presented has a normal distribution and was analyzed by One-way ANOVA with a post hoc Tukey test. The myofibril quantification data and the flight test data were not normally distributed and was therefore analyzed with the Kruskal-Wallis One-way ANOVA with a post hoc all pairwise multiple comparison pooled sample median test. For all statistical tests, p<0.05 was considered statistically significant.

## Results

### Decreasing Mio levels increases storage of glycogen in muscle cells

**Fig 1:**
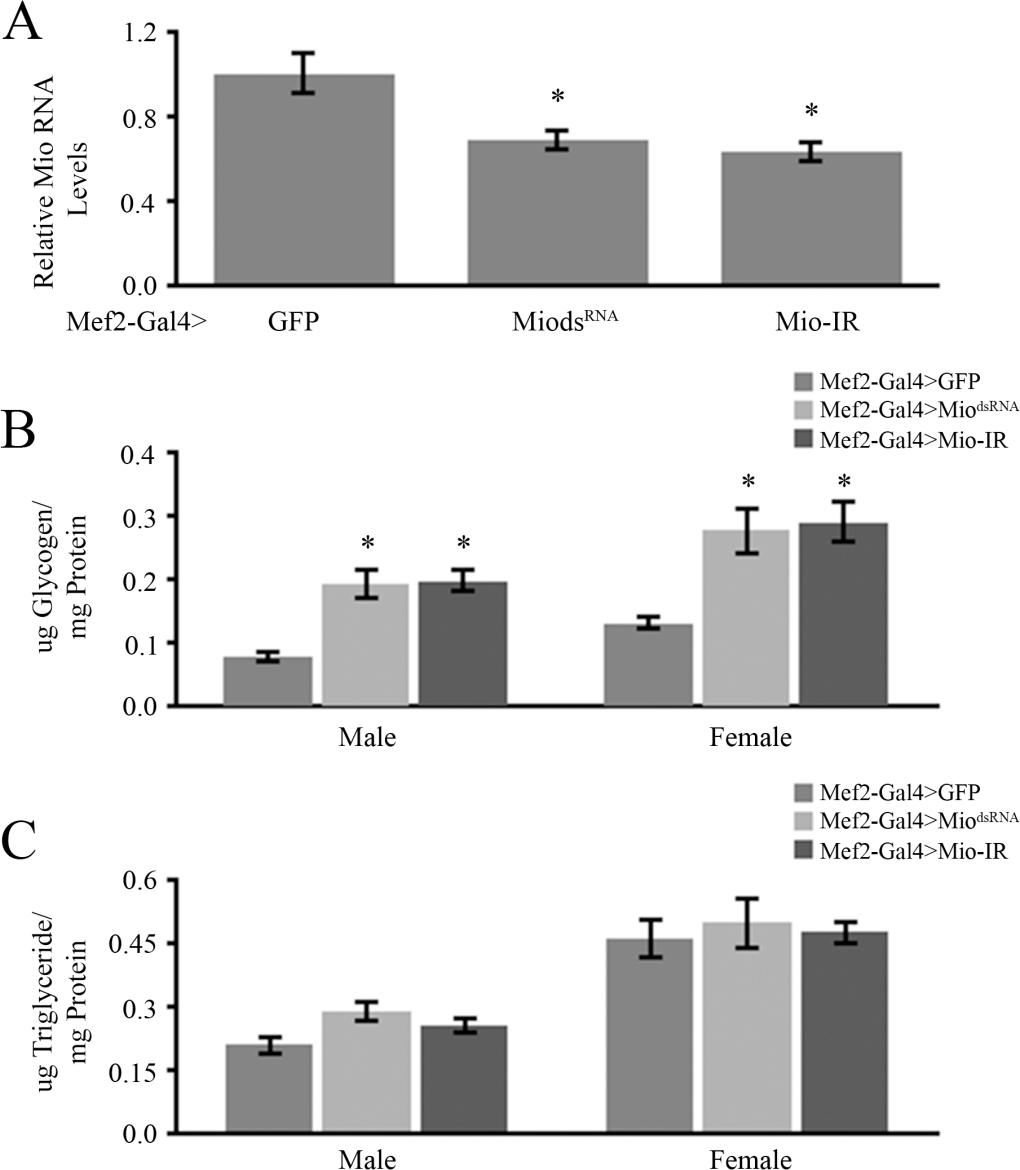
Mio expression in muscle is necessary for normal glycogen storage. (A) Analysis of Mio expression from thoraxes of 5–7 day old Mef2-Gal4>Mio^dsRNA^ and Mef2-Gal4>Mio-IR flies compared to Mef2-Gal4>GFP controls (n = 8). Mio levels of the Mef2-Gal4>GFP controls were set to 1.0 and Mio levels of Mef2-Gal4>Mio^dsRNA^ and Mef2-Gal4>Mio-IR animals were then calculated relative to their respective control. Values represent mean *Mio* expression±SEM. *p<0.05 by One-way ANOVA with post hoc Tukey test. (B) Glycogen/protein and (C) triglyceride/protein of thoraxes dissected from 5–8 day old Mef2-Gal4>Mio^dsRNA^ and Mef2-Gal4>Mio-IR male and female flies compared to their respective Mef2-Gal4>GFP controls (n = 6). Values represent mean±SEM. *p<0.05 by One-way ANOVA with post hoc Tukey test.

Previous studies have shown that Mio expression in the fat body promotes triglyceride storage in *Drosophila* larvae and adults [18–20]. Mio is also expressed in other tissues [18] but its functions there remain unknown. In order to understand the tissue-specific functions of Mio, we decided to focus on *Drosophila* muscle, a highly metabolic and physiologically important tissue. To characterize the role of Mio in muscle, we performed experiments in which RNA interference (RNAi) was used to decrease *Mio* expression specifically in all muscles using Mef2-Gal4 [32] (Fig 1A). In order to achieve the desired knockdown we used two independent RNAi lines that provide lower levels of Mio. Mio^dsRNA^ uses double stranded RNA in order to activate the RNAi machinery while Mio-IR uses inverted repeats to activate RNAi. Using two independent RNAi transgenes allows us to control for any potential off target effects of the RNAi as well as any potential transgene insertion effects on the data presented here. Since Mio is important for triglyceride storage in the fat body and Mio’s mammalian homolog ChREBP has been implicated in the regulation of triglyceride and glycogen storage in liver and adipose tissue [10], we wanted to determine whether Mio acts in muscle to regulate macromolecule storage. To test this hypothesis, glycogen and triglyceride levels were measured from thoraxes of muscle-specific Mio-RNAi flies. Thoraxes from flies with decreased *Mio* levels in all muscles showed significantly higher glycogen levels when compared to GFP controls while no differences were observed in triglycerides (Fig 1B and 1C), suggesting that Mio plays a role specifically in glycogen storage in muscle.

### Mio is necessary for normal flight

**Fig 2:**
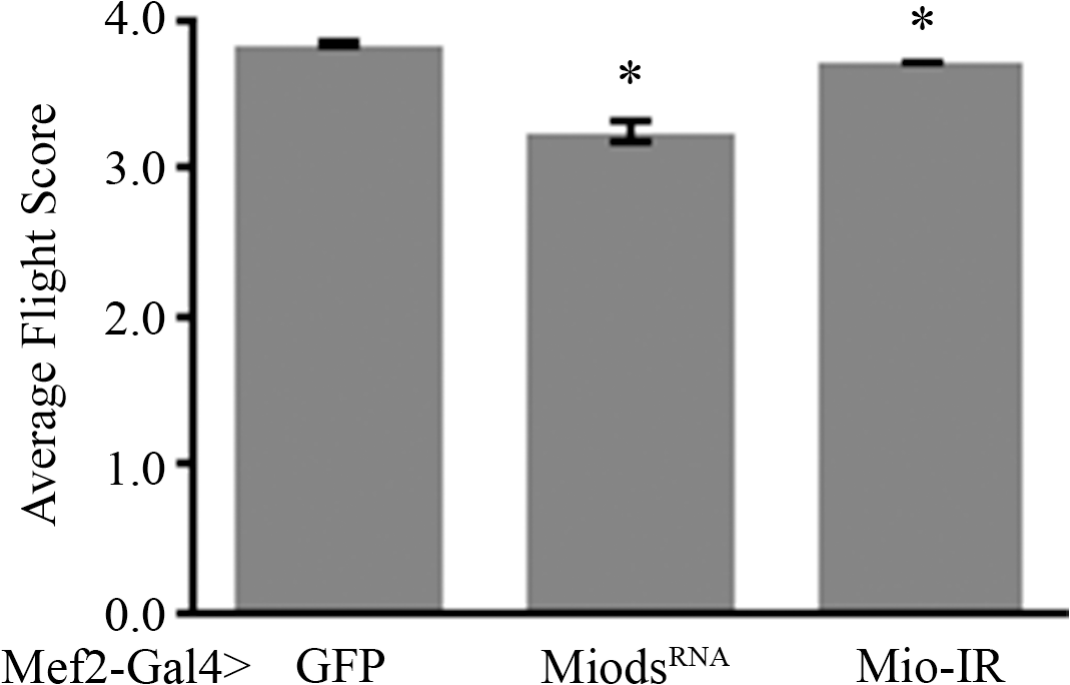
Mio in muscle is necessary for normal flight. Flight tests were performed on Mef2-Gal4>Mio^dsRNA^ (n = 144) and Mef2-Gal4>Mio-IR (n = 296) flies and compared to Mef2-Gal4>GFP (n = 179) controls and scored based on flight ability (See methods). Values represent average flight score ±SEM. *p<0.05 Kruskal-Wallis One-way ANOVA with post hoc all pairwise multiple comparison pooled sample median test.

Since flies with decreased *Mio* levels accumulated glycogen in their muscle cells, we wanted to know whether this had any effect on the function of these muscles. Muscle-specific Mio-RNAi flies showed no gross problems associated with wing or body structure when compared to the controls and their walking ability was not impaired, suggesting that loss of Mio had little effect on these muscle groups. Because Mio-RNAi flies showed no gross problems associated with movement or body structure, we turned our attention to another muscle group that has been well characterized in the *Drosophila* adult, the flight muscles. To determine whether decreased levels of Mio had any effect on flight muscle function, we measured the flight ability of Mio-RNAi flies. Flies with decreased *Mio* expression showed a significant decrease in flight ability when compared to the control flies (Fig 2), suggesting a flight muscle-specific function of Mio. These data indicate that Mio is playing a role in the normal function of flight muscles in *Drosophila*.

### Mio is important for myofibril ultrastructure

**Fig 3:**
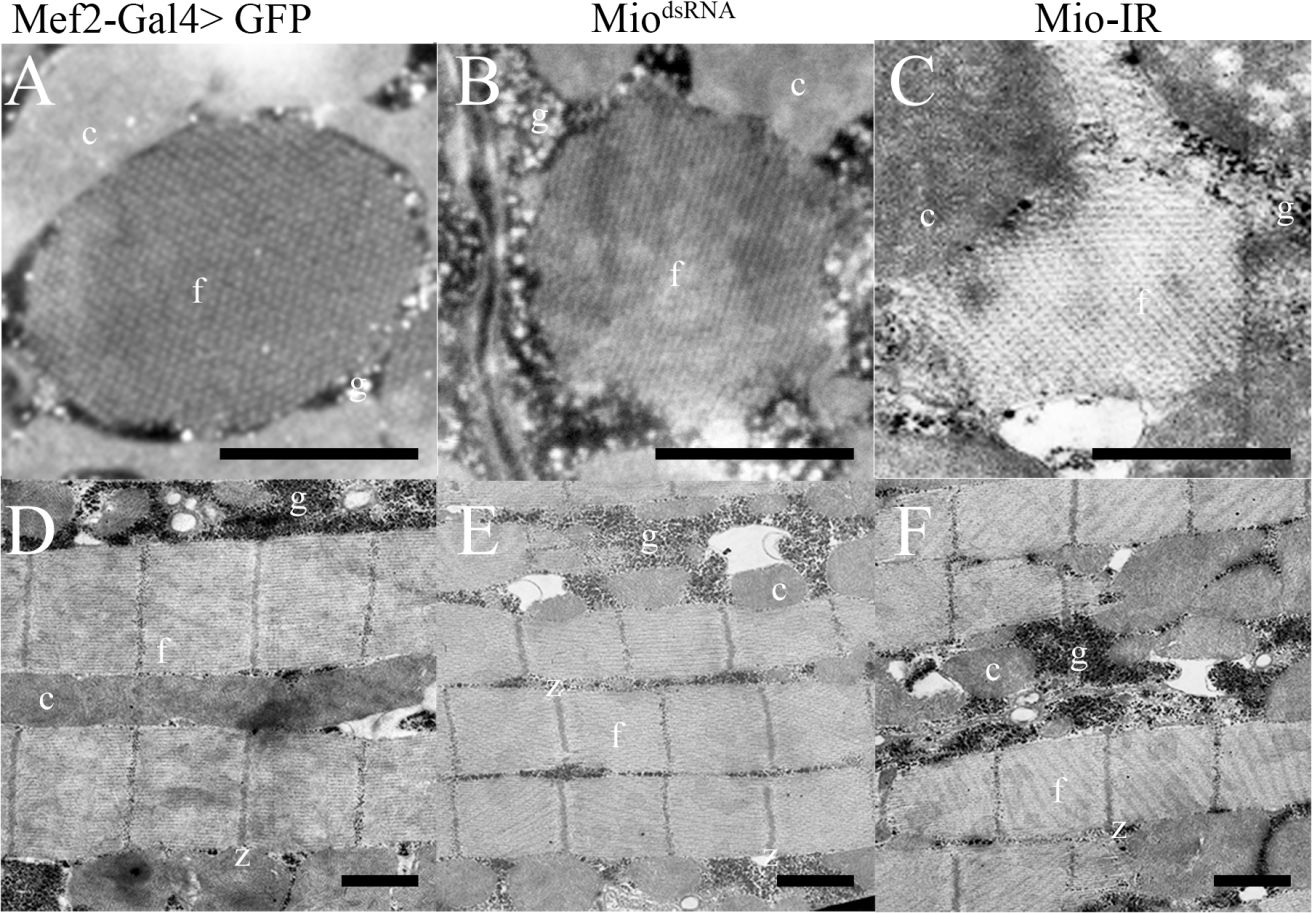
Decreasing Mio levels results in abnormal myofibril ultrastructure. Transmission Electron Microscopy of Indirect Flight Muscles of adult Mef2-Gal4>Mio^dsRNA^ and Mef2-Gal4>Mio-IR females compared to Mef2-Gal4>GFP controls. Panels (A), (B) and (C) show cross sections of the myofibrils; panels (D), (E) and (F) show longitudinal sections of myofibrils. f, myofibril; c, mitochondrion; g, glycogen granules; m, m-line; z, z-line. Scale bar = 0.5μm.

The phenotypes observed in many previously identified *Drosophila* flight mutants have resulted from altered myofibril structure [22]. To test whether loss of Mio results in flight impairment due to altered muscle structure, we made use of Transmission Electron Microscopy (TEM) to visualize the indirect flight muscles (IFM). Micrographs of IFM ultrastructure showed abnormal myofibril assembly in cross sectional views in adult flies where *Mio* was decreased when compared to their GFP-expressing controls. Cross sections of Mio-RNAi myofibrils revealed normal actin/myosin crystal lattice structure, but the myofibrils themselves failed to retain their circular shape and looked smaller than the control myofibrils (Fig 3A–3C). IFM from Mio-RNAi flies also visually accumulated more glycogen granules around the myofibrils when compared to controls (Fig 3A–3C), consistent with the increased glycogen phenotype observed above. Although cross sections of Mio-RNAi flies showed disrupted myofibril ultrastructure, longitudinal sections revealed normal sarcomere assembly (Fig 3E and 3F) when compared with their respective GFP controls (Fig 3D). In addition, no obvious alterations in mitochondrial morphology were observed in muscles from Mio-RNAi flies (unpublished observations) suggesting that Mio does not regulate mitochondrial structure.

**Fig 4:**
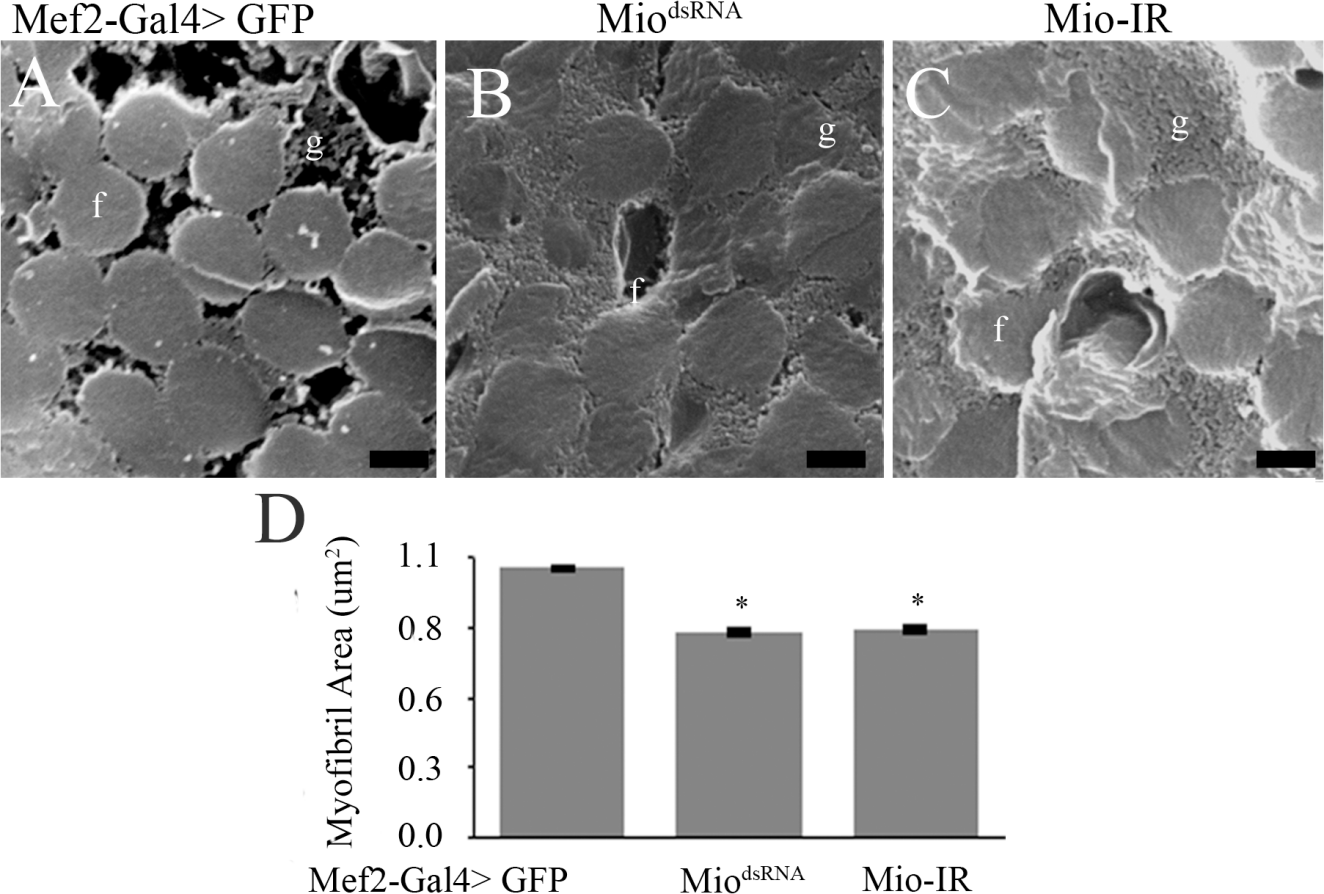
Mio affects myofibril size. Scanning Electron Microscopy of Indirect Flight Muscles of adult Mef2-Gal4>Mio^dsRNA^ and Mef2-Gal4>Mio-IR females compared to Mef2-Gal4>GFP controls. Panels (A), (B) and (C) show cross sections of the myofibrils; bars indicate 0.5μm. f, myofibril; c, mitochondrion; g, glycogen granules. (D) Average myofibril area of Mef2-Gal4>Mio^dsRNA^ and Mef2-Gal4>Mio-IR flies compared to Mef2-Gal4>GFP control flies (n = 3–5). All values represent average myofibril area ±SEM. *p<0.05 Kruskal-Wallis One-way ANOVA with post hoc all pairwise multiple comparison pooled sample median test.

**Fig 5:**
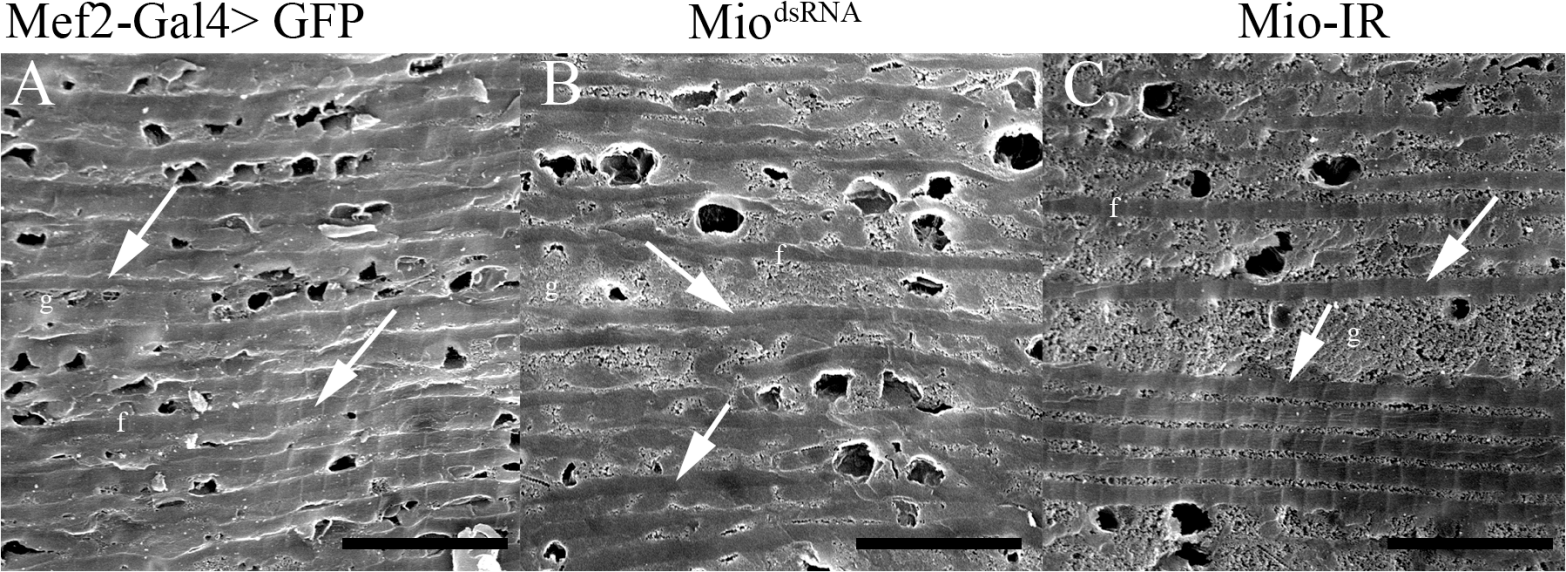
Decreasing Mio levels results in abnormal myofibril organization. Scanning Electron Microscopy of longitudinal sections of Indirect Flight Muscles of adult Mef2-Gal4>Mio^dsRNA^ and Mef2-Gal4>Mio-IR females compared to Mef2-Gal4>GFP controls. f, myofibril; g, glycogen granules. Each arrow points to a single longitudinal myofibril. Scale bar = 20μm.

In order to confirm the small myofibril phenotype observed in the TEM micrographs, Scanning Electron Microscopy (SEM) of IFMs was performed. Cross-sections observed under SEM also showed incorrect assembly of myofibrils where individual myofibrils lacked a defined circular/oval shape as seen in the control flies (Fig 4A–4C), consistent with the TEM data shown above. Quantitation of cross-sectional myofibril area in flies with decreased *Mio* also showed smaller myofibril size when compared to controls (Fig 4D). In addition to the smaller myofibril phenotype, longitudinal sections of myofibrils from animals where *Mio* levels were decreased showed irregularly spaced myofibrils filled with glycogen granules (Fig 5B and 5C), which is in contrast to the normal myofibril-mitochondria-myofibril pattern that was observed in the control muscles (Fig 5A). Together, these data suggest that Mio is important for muscle structure.

**Fig 6:**
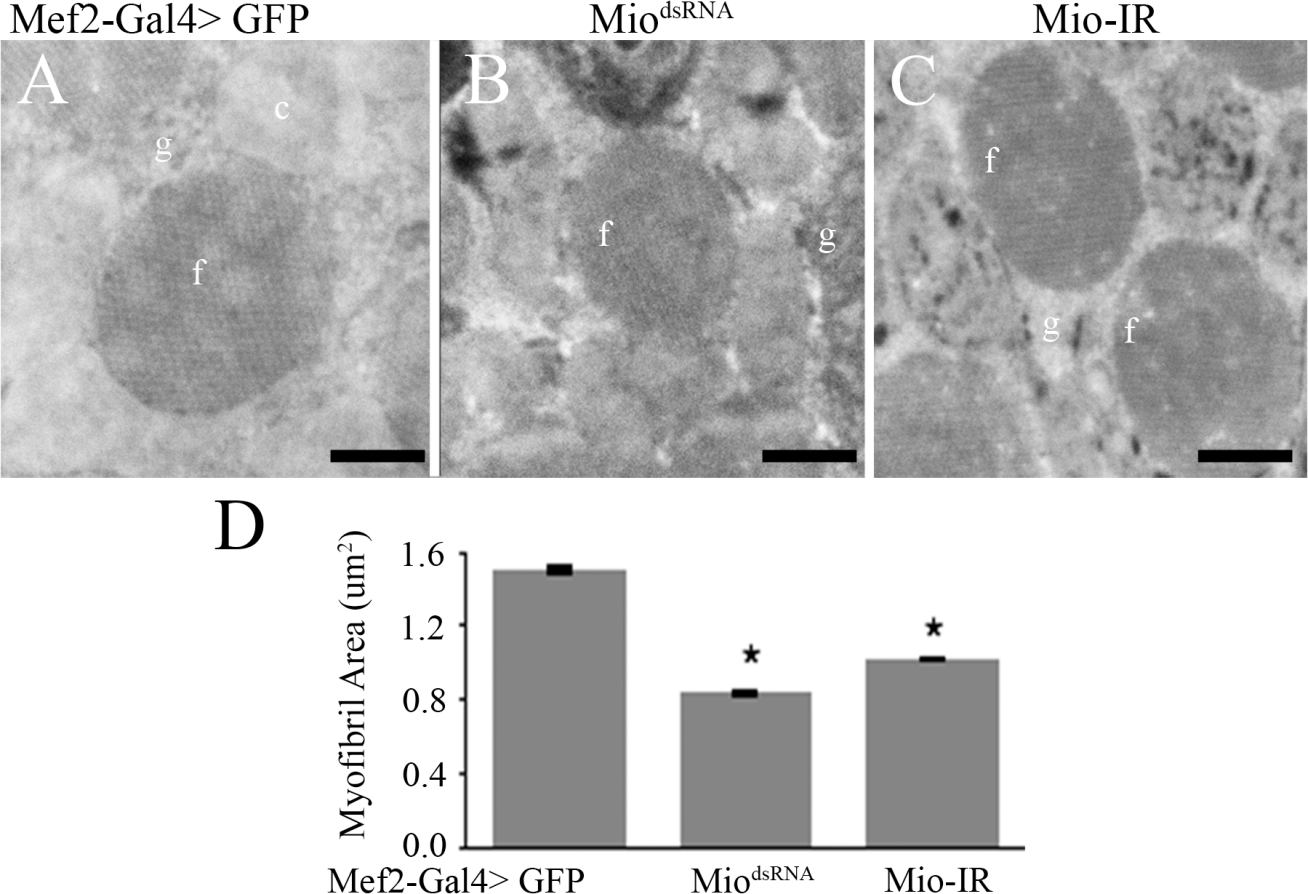
Mio affects myofibril size in pharate adults. Transmission Electron Microscopy of Indirect Flight Muscles of Mef2-Gal4>Mio^dsRNA^ and Mef2-Gal4>Mio-IR pharate adults compared to Mef2-Gal4>GFP controls. Panels (A), (B) and (C) show cross sections of the myofibrils. Bars indicate 0.5μm. f, myofibril; c, mitochondrion; g, glycogen granules. (D) Average myofibril area of Mef2-Gal4>Mio^dsRNA^ and Mef2-Gal4>Mio-IR pharate adults compared to Mef2-Gal4>GFP controls (n = 3–5). Values represent average myofibril area ±SEM. *p<0.05 by One-way ANOVA with post hoc Tukey test.

To determine whether the altered myofibril phenotype in adult flies was generated by muscle usage or due to a defect in myofibril assembly during development, we visualized the IFMs in pharate adults at the end of the pupal stage of development just before emerging from the pupal case. TEM micrographs of the IFM showed normal assembly of myofibrils in the Mio-RNAi flies with normal crystal lattice formation and round, normally shaped myofibrils (Fig 6A–6C). Although we did not detect any structural problems in myofibrils from Mio-RNAi pharate adults, we did observe smaller cross-sectional areas of myofibrils in animals with decreased Mio levels compared to the GFP controls (Fig 6D), consistent with the smaller myofibrils in Mio-RNAi adults (Fig 4D). These data indicate that Mio might be playing a role in the development and/or assembly of myofibrils in addition to its metabolic function in muscle cells.

**Fig 7:**
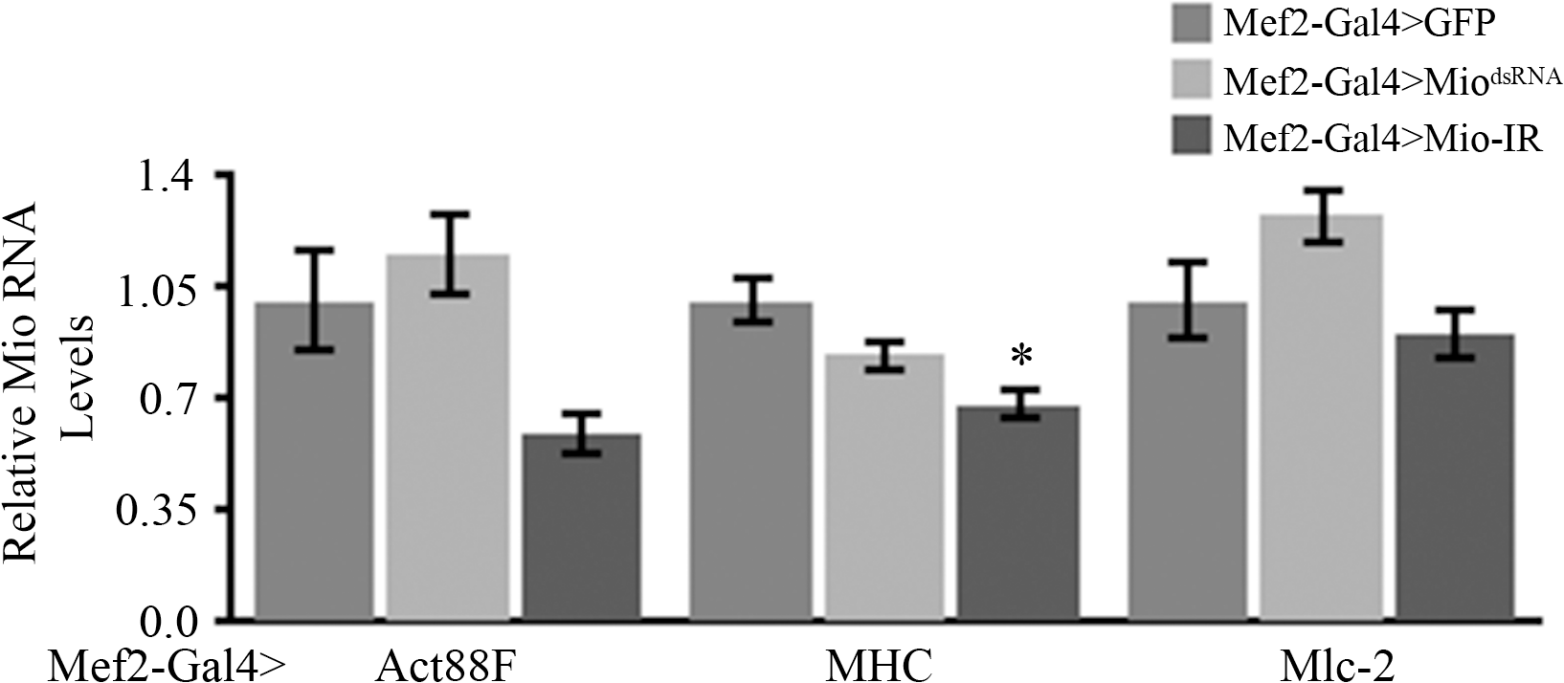
Mio is not necessary for normal expression of structural genes in adult fly muscle. Expression of Actin 88F (Act88F, n = 8), Myosin Heavy Chain (MHC, n = 11), and Myosin light chain-2 (Mlc-2, n = 8) was measured by performing quantitative PCR on thorax cDNA from 5–7 day old Mef2-Gal4>Mio^dsRNA^ and Mef2-Gal4>Mio-IR flies and compared to Mef2-Gal4>GFP controls. mRNA levels of Mef2-Gal4>GFP controls were set to 1.0 and mRNA levels of Mef2-Gal4>Mio^dsRNA^ and Mef2-Gal4>Mio-IR animals were then normalized to their respective control. Values represent mean±SEM. *p<0.05 by One-way ANOVA with post hoc Tukey test.

To test this hypothesis, we measured the mRNA levels of an indirect flight muscle-specific actin gene (*Act88F)*, Myosin heavy chain (*Mhc)*, and Myosin light chain-2 *(Mlc-2)*, genes important for muscle development and assembly that when mutated cause a similar altered myofibril phenotype as seen in our *Mio-*deficient animals [21, 33–35]. Interestingly, no consistent differences in the expression of these genes were observed in Mio-RNAi adults (Fig 7). Together, these data suggest that *Mio* does not function to regulate the expression of genes required for myofibril assembly, but may be important for normal metabolic homeostasis of muscle cells throughout the life cycle of the animal.

## Discussion

In this study, we have shown that decreasing *Mio* levels specifically in *Drosophila* muscle results in the accumulation of glycogen and leads to flight impairment due to altered myofibril ultrastructure. Early genetic screens performed in *Drosophila melanogaster* identified many genes that when mutated cause flightless or limited flight phenotypes as well as irregular structures of the DFM and/or IFM [24, 36, 37]. Mutations in myofibril structural genes, such as myosin heavy chain (Mhc) and myosin light chain-2 (Mlc-2), result in moderate to extreme disorganization of the myofibril at its periphery and the inability to maintain the circular shape of the sarcomere [24, 34, 35, 37]. *Mhc* and *Mlc-2* mutants also have smaller myofibrils and sarcomeres that are disorganized at the periphery, but maintain their crystal lattice structure [34, 35]. Decreasing *Mio* in muscle results in a very similar myofibril structure phenotype with smaller but misshapen myofibrils (Figs 3 and 4). Therefore, it is possible that Mio may also play a role in regulating muscle formation during development. To address the contribution of Mio to myofibril development and assembly in IFMs, we examined the structure of the IFM myofibrils in pharate adults at the end of the pupal stage of development before the flies eclosed from their pupal cases and started using their IFMs. Interestingly, myofibrils from pharate adult IFMs with decreased Mio assemble correctly and their myofibrils are oval in shape like the control myofibrils. However, the size of the myofibrils is decreased in Mio-RNAi animals. In contrast to the smaller, but normal shaped myofibrils in IFMs from pharate adults, myofibrils from adult flies with decreased Mio levels were small as well as misshapen (Figs 3 and 4). This suggests that the increased age of the adult flies or the usage of the IFMs between emerging from the pupal cases and when the muscles were collected for analysis may account for the additional altered shape phenotype observed in Mio-RNAi adult flies. In addition, despite the fact that *Mhc* and *Mlc-2* mutants have similar myofibril structural phenotypes to Mio-RNAi flies [33–35], Mio does not seem to regulate the expression of these important structural genes, as no consistent changes in *Mhc*, *Mlc-2*, and *Act88F* mRNA levels were observed with both RNAi transgenes (Fig 7). However, whether Mio regulates other genes important for muscle development and function is not known. Determining the full complement of Mio target genes and observing myofibril structure at additional timepoints in the lifespan of the fly will be important to understand the role Mio plays in the development and assembly of myofibrils.

The accumulation of glycogen itself may also be responsible for the muscle atrophy observed in the Mio-RNAi flies. Glycogen phosphorylase deficiency in humans leads to the depletion of ATP, as glycogen cannot be utilized for energy during muscle contraction, resulting in increased glycogen deposition and muscle atrophy [38, 39]. Increased storage of glycogen in muscle has also been seen in mammals in a disease known as polysaccharide storage myopathy (PSSM). PSSM has been observed in horses where glycogen molecules accumulate within the muscle, leading to weakness and ultimately muscular atrophy [40, 41]. In all PSSM cases, excess glycogen accumulation can be counteracted by changes in diet, and this then reduces the effects of PSSM and prevents muscular atrophy. Whether the increased glycogen levels observed in the Mio-RNAi flies is responsible for the muscle structure phenotypes observed in these animals is still unknown. Future experiments on flies with excess glycogen stores could help to understand the role of glycogen accumulation on muscular atrophy.

The altered muscle structure and function phenotypes observed here are reminiscent of muscle wasting observed in cancer patients (cachexia) and the aging (sarcopenia) [4, 42]. Several studies have shown that decreasing activity through the insulin/insulin-like growth factor 1 (IGF-1) signaling pathway (either genetically or due to tumor-based production of the insulin/IGF antagonist *ImpL2*) leads to muscle wasting and affects genes encoding metabolic enzymes as well as macromolecule stores [43–47]. Interestingly, previous studies in both mammals and flies suggest that ChREBP and Mio are transcriptional targets of insulin [48, 49]. Therefore, it is possible that ChREBP and Mio are functioning with the insulin/IGF-1 pathway to regulate muscle structure and function in both normal and diseased states.

## Conclusions

In summary, the data presented in this study identifies a novel role of Mio, the *Drosophila* homolog of mammalian ChREBP and MondoA, in muscle tissue. In addition to its known role in metabolism, we show that Mio is important for the structure and metabolism of myofibrils, providing a link between nutrient availability and muscle function. This study also highlights the utility of the *Drosophila* system for identifying and understanding genes important for regulating muscle metabolism and physiology.
